# TCP1 regulates Wnt7b/β-catenin pathway through P53 to influence the proliferation and migration of hepatocellular carcinoma cells

**DOI:** 10.1038/s41392-020-00278-5

**Published:** 2020-08-25

**Authors:** Nanhong Tang, Xiaoling Cai, Lirong Peng, Hekun Liu, Yuanzhong Chen

**Affiliations:** 1 Department of Hepatobiliary Surgery and Fujian Institute of Hepatobiliary Surgery, Fujian Medical University Union Hospital, Fuzhou, China; 2 Fujian Key Laboratory for Translational Research in Cancer and Neurodegenerative Diseases, Institute for Translational Medicine, Fujian Medical University, Fuzhou, China; 3 Fujian Institute of Hematology, Fujian Provincial Key Laboratory on Hematology, Fujian Medical University Union Hospital, Fuzhou, China

**Keywords:** Prognostic markers, Gastrointestinal cancer

**Dear Editor,**

Molecular chaperones are critical mediators of oncogenesis and necessary for cell survival.^1^ The cytoplasmic chaperone TRiC (T-complex protein-1 ring complex, also known as CCT) comprises two back-to-back stacked rings, with each ring containing eight subunits (CCT1–CCT8).^2^ And the effects of CCT subunits on tumors may be different. However, the roles of the CCT1 subunit (also known as TCP1) in hepatocellular carcinoma (HCC) and its molecular mechanism have not been investigated thoroughly. Understanding these details can provide new ideas and strategies for treating HCC.

In this study, we found that the level of TCP1 in HCC was significantly higher than that in the corresponding adjacent tissues, and a higher TCP1 expression was observed in poorly differentiated liver tumors (Supplementary Fig. S1a, b). Meanwhile, compared with patients with a low expression of TCP1, those patients with a high expression of TCP1 had a shorter total survival time, or disease-free survival and higher hazard ratio (Fig. 1a). Furthermore, The TCGA and Oncomine database analysis showed that the expression of TCP1 in HCC was significantly higher than that in normal liver tissue (Supplementary Fig. S1c, d). The Kaplan–Meier survival analysis showed that among HCC patients in clinical stages III/IV and pathological stages G3/G4, those with a high expression of TCP1 had a worse survival status (Supplementary Fig. S1e). Therefore, high expression of TCP1 is related to poor survival and prognosis in HCC.

**Fig. 1:**
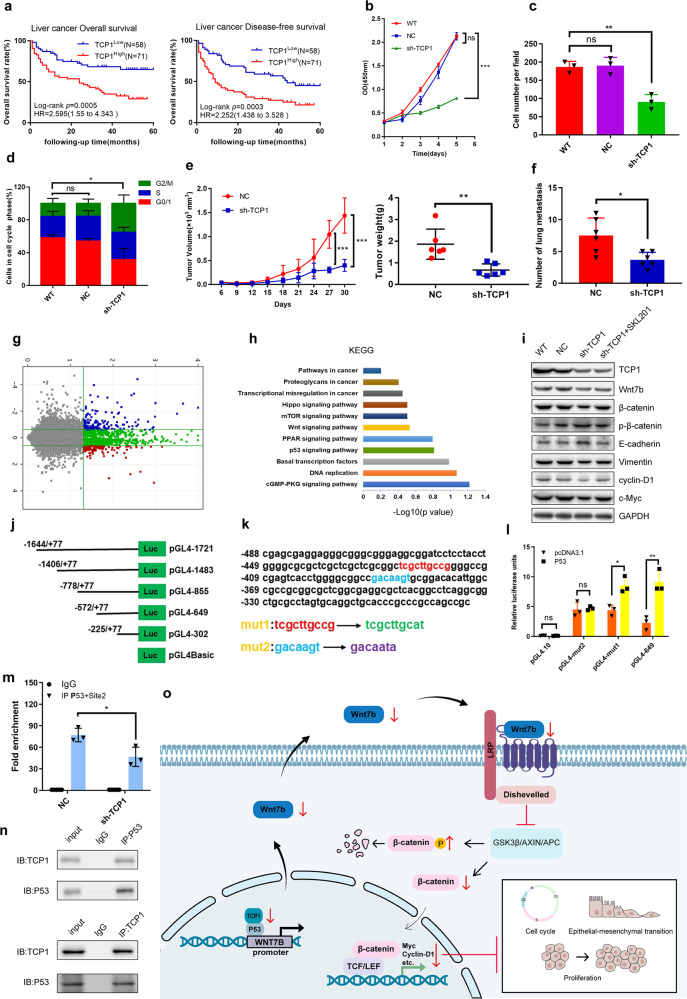
TCP1 regulates Wnt7b/β-catenin pathway through P53 to influence the proliferation and migration of hepatocellular carcinoma cells. **a** Kaplan–Meier analysis of overall survival and disease-free survival of liver cancer patients. **b** Cell growth was measured using the CCK-8. **c** Transwell migration assay. **d** Cells were stained with PI for cell cycle analysis. **e** The tumor growth curves and the tumor weight of NC and sh-TCP1 mice. **f** The numbers of lung metastasis. **g** The differential gene expression profiles. **h** The significant signaling pathways of the differentially expressed genes. **i** Western blot analysis to detect the protein expression of the Wnt pathway. **j** Multiple luciferase report plasmids of the WNT7B promoter. **k**, **l** Potential P53 binding sites in the WNT7B promoter core region. **m** ChIP-qPCR. **n** CoIP analyze the interaction between TCP1 and P53. **o** TCP1 interacts with P53 to regulate Wnt7b/β-catenin pathway. Data represent the mean ± SEM. *P < 0.05, **P < 0.01, and ***P < 0.001; ns no significance

To explore the biological function of TCP1 in HCC, we detected the effect of TCP1 in HCC cell lines and animal models. The analysis showed that the downregulation of TCP1 expression can significantly reduce cell viability, inhibited cell proliferation, and migration, the cell cycle was blocked in the G2/M phase in vitro (Fig. 1b–d and Supplementary Fig. S2a–d). In addition, xenograft models indicated that the knockdown of TCP1 inhibited the growth of subcutaneous implanted tumor (Fig. 1e and Supplementary Fig. S2e) and lung metastasis in vivo (Fig. 1f and Supplementary Fig. S2f, g). Previous studies have found that CCT and other driving gene combinations jointly mediate the proliferation of cancer cells, and that TCP1 plays an important role in lymph tumor metastasis.^3^ The influence of TCP1 on the phenotype of HCC cells is consistent with that on other types of tumors.

Next, we investigated the mechanism behind the biological function change of HCC cells, as induced by TCP1 knockdown. A high-throughput whole-genome expression chip was used to analyze the gene expression difference between sh-TCP1 (knockdown of TCP1) and NC (negative control). A total of 177 differentially expressed genes (foldchange > 1.5, *P* < 0.05) were observed, 101 of that were upregulated and 76 were downregulated (Fig. 1g and Supplementary Fig. S3a). We further analyzed the co-expression of TCP1 and these differentially expressed genes by using data from the GEPIA (Gene Expression Profiling Interactive Analysis) database (Supplementary Table 1). The KEGG pathway analysis showed that the differentially expressed gene Wnt7b participated in many important signaling pathways, including Wnt, mTOR, Hippo, proteoglycans in cancer, and pathways in cancer signaling pathway, the difference of the Wnt signaling is the most significant among these pathways (Fig. 1h and Supplementary Fig. S3b). We detected the expression of key molecules in the Wnt signaling pathway targeting Wnt7b. Western blot showed that the downregulation of TCP1 significantly reduced the expression of Wnt7b and β-catenin, and increased the expression of p-β-catenin. However, the expression of JNK and p-JNK were not significantly changed (Supplementary Fig. S3c, d). We further detected that the protein expression was related to the epithelial mesenchymal transformation, migration, and cell cycle in downstream of the Wnt signaling pathway. The downregulation of TCP1 significantly increased the expression of E-cadherin and reduced the expression of vimentin, c-Myc, and cyclin-D1. After the treatment with SKL2001 (a Wnt pathway agonist targeting β-catenin), the protein levels of p-β-catenin, E-cadherin were significantly downregulated, and β-catenin, vimentin, c-Myc, and cyclin-D1 were significantly upregulated in sh-TCP1 group (Fig. 1i and Supplementary Fig. S3e, f). These results suggested that TCP1 knockdown can prevent the activation of the Wnt/β-catenin signaling pathway through Wnt7b, thereby inhibiting the proliferation and migration of HCC cells. We highlighted the importance and value of this pathway as a target therapy, but the toxicity of Wnt inhibitors may not be tolerated by HCC patients with limited capacity for liver cell regeneration.^4^ Therefore, choosing the upstream target of Wnt for intervention may present a new direction in clinical liver cancer treatment research.

To further explore the molecular mechanism of TCP1 regulation in Wnt7b, we used the PROMO and JASPAR databases to predict the transcription factors involved in the regulation of the WNT7B promoter. Following the previous finding of an interaction between TCP1 and P53,^5^ we used TFBIND to predict the binding site of P53 in the WNT7B promoter (Supplementary Table 2) and to determine whether TCP1 interacts with P53 to regulate Wnt7b expression. We constructed several luciferase reporter plasmids based on the predictions (Fig. 1j). The double luciferase report analysis showed that the pGL4-302 (−225 to +77) fragment had no significant luciferase activity, whereas pGL4-649 (−572 to +77) had a relatively strong luciferase activity. In addition, the co-transfection of the P53 expression vector with promoter fragment showed that the P53 expression significantly enhanced the luciferase activities induced by the WNT7B promoter, including pGL4-1721, pGL4-1483, pGL4-855, and pGL4-649, indicating that the key active region of the WNT7B promoter might be located from −572 to −225 (Supplementary Fig. S4a–c). Then, we constructed mutant plasmids (mutations 1 and 2) at these binding sites (Fig. 1k). Mutation 2 significantly reduced the transactivation of P53 to WNT7B (Fig. 1l and Supplementary S4d). The ChIP assay showed that P53 can be enriched to the chromatin fragment containing site 2 and that TCP1 knockdown significantly reduced the enriched fragment (Fig. 1m). The CoIP assay suggested a protein–protein interaction between TCP1 and P53 (Fig. 1n), and overexpression of P53 significantly improved the result of low wnt7b expression caused by TCP1 knockdown (Supplementary Fig. S4e), thereby indicating that P53 can bind to WNT7B promoter site 2 and that TCP1 affects the expression of Wnt7b by interacting with P53. Furthermore, we used GEPIA database to analyze the relationship among TCP1, p53, and WNT7B in HCC (LIHC), and found that TCP1 is significantly associated with p53 and WNT7B, p53 is also obviously correlated with WNT7B (Supplementary Fig. S4f).

In summary, we found an important role for TCP1/Wnt7b/β-catenin in promoting HCC cell proliferation and metastasis through P53 (Fig. 1o), and TCP1 may be a valuable molecular marker for the diagnosis and prognosis of HCC. This study also provides theoretical and experimental basis for the drug research targeting TCP1 in the treatment of HCC.

## Supplementary information

Supplementary Materials
