# Supplementary material for: TCP1 regulates Wnt7b/β-catenin pathway through P53 to influence the proliferation and migration of hepatocellular carcinoma cells
Source: Signal Transduct Target Ther. 2020 Aug 25;5:169. doi: 10.1038/s41392-020-00278-5 (PMC7447807; doi:10.1038/s41392-020-00278-5)
Supplement: Supplementary file 1 — Supplementary Materials [file 41392_2020_278_MOESM1_ESM.docx]

Supplementary Materials for

**TCP1 regulates Wnt7b/β-catenin pathway through P53 to influence the proliferation and migration of hepatocellular carcinoma cells**

Nanhong Tang^1^, Xiaoling Cai^1^, Lirong Peng^1^, Hekun Liu^2^, Yuanzhong Chen^3^

*^1^Department of Hepatobiliary Surgery and Fujian Institute of Hepatobiliary Surgery, Fujian Medical University Union Hospital, Fuzhou, China; ^2^Fujian Key Laboratory for Translational Research in Cancer and Neurodegenerative Diseases, Institute for Translational Medicine, Fujian Medical University, Fuzhou, China and ^3^Fujian Institute of Hematology, Fujian Provincial Key Laboratory on Hematology, Fujian Medical University Union Hospital, Fuzhou, China*

*These authors contributed equally: Nanhong Tang, Xiaoling Cai*

*Correspondence: Yuanzhong Chen (chenyz@mail.fjmu.edu.cn)*

**This file includes:**

Materials and Methods  **2**

Supplemental Figures **5**

Fig. S1 TCP1 expression is elevated in HCC tissues and correlates with a poor prognosis 5

Fig. S2 Knockdown of TCP1 inhibits the proliferation and migration of HCC *in vitro* and *in vivo* 6

Fig. S3 Downregulated TCP1 expression inhibits the Wnt/β-catenin signaling pathway of HCC cells through Wnt7b 7

Fig. S4 TCP1 regulates WNT7B transcription by binding transcription factor P53 in HCC cells 9

Supplemental Tables **10**

Supplementary Table 1. List of genes affected by TCP1 knockdown and Co-expressed With TCP1 10

Supplementary Table 2. List of transcription factors predicted by PROMO and JASPAR 11

Supplementary Table 3. Antibodies used in the present study 11

Supplementary Table 4. List of oligonucleotides used in this study 12

**Materials and Methods**

**Patients and specimens**

A total of 156 paraffin samples of HCC were obtained from FJMU Union hospital and First affiliated hospital of FJMU. 27 of the cancer tissue sections of HCC compared with corresponding paracancerous tissue (Dataset 1) were evaluated the expression level of TCP1 by semiquantitatively analyzing using the following equation: Mean Optical Density (MOD) = Integral Optical Density (IOD)/Positive Area. All image analyses were conducted using Image-Pro Plus 6.0 software (Media Cybernetics, Rockville, MD). The remaining 129 pathological specimens (Dataset 2) were divided into poorly differentiated (PD, N=87), moderately differentiated (MD, N=30), and well differentiated (WD, N=12) according to the degree of differentiation, and follow-up data were collected. These sections were examined and scored independently by two investigators in a double-blinded manner. The staining intensity was determined according to a histological scoring method. The histological score for each section was computed using the following formula: histological score = proportion score × intensity score. A total score with a possible range of 0–12 was calculated and graded as follows: negative (−, score: 0), weak (+, score: 1–4), moderate (++, score: 5–8) or strong (+++, score: 9–12). Scores of “−” and “+” were considered to indicate low expression levels, whereas scores of “++” and “+++” were considered to indicate high expression levels.

**Immunohistochemistry (IHC)**

Clinical specimens were examined using immunohistochemistry staining. After formalin fix and paraffin embedment, tissue sections were deparaffinized in dimethylbenzene, and rehydrated in alcohol and water. Endogenous peroxidase activity was blocked with 3% hydrogen peroxide. Antigen retrieval was performed by heat treatment. Nonspecific binding was blocked with 10% BSA (Sigma). Primary antibodies against TCP1 (1:400, Abcam) were incubated with the sections overnight at 4°C, followed by incubated with horseradish peroxidase-conjugated secondary antibody for 30 min at 37°C. Immunocomplexes in sections were stained with 3,3-diaminobenzidine (Sigma) to form brown reaction products. After counterstaining with hematoxylin, sections were dehydrated and mounted for microscopic examination.

**Database analysis**

Liver cancer and normal cell gene expression data and clinical data, including tumor stage and overall survival, were downloaded from The Cancer Genome Atlas (TCGA) database (https://cancergenome.nih.gov/).Gene expression data were available for 371 HCC patients. Expression levels were log2 transformed. For the survival analyses, the group cutoff was set to median (cutoff‐High and cutoff‐Low are both 50%), HCC patients with low expression levels of TCP1 (the lower 50%, n=185) were compared with the high expression levels (the upper 50%, n=185). The survival data were analyzed by using the Kaplan–Meier method. The Oncomine database (https://www.oncomine.org/resource/login.html) obtained mRNA expression of TCP1 in liver cancer tissues and normal tissues.

**Cell culture and transfection**

MHCC-97H (Fudan University Hepatology Research Institute, Shanghai, China) and HepG2 (Shanghai Cell Biology Institute of Chinese Academy of Science, Shanghai) were maintained in Dulbecco’s modified Eagle medium (DMEM, Gibco, Carlsbad, CA) supplemented with 12% foetal bovine serum (FBS, Gibco). Synthesized short hairpin RNA oligonucleotides targeting TCP1 (sh-TCP1) and negative control short hairpin RNA oligonucleotides (NC) were annealed and ligated into the pLent-U6-GFP-Puro lentiviral vector (Genechem, Shanghai). MHCC-97H and HepG2 cells were used to infect according to the manufacturer’s recommendations. Puromycin (2 μg/ml final concentration for HepG2 and 1.5 μg/ml for MHCC-97H) was used to select for stably infected cells.

**Western blot assay**

Equivalent amounts of protein were applied to 12% or 10% sodium dodecyl sulphate-polyacrylamide gel electrophoresis (SDS-PAGE) and electrophoretically transferred to a polyvinylidene fluoride (PVDF) membrane (Merck Millipore, Darmstadt, Germany). Western blots were incubated separately f­rom a panel of specific antibodies (Supplementary Table 3). Immunoreactivity was detected using a chemiluminescent immunoblot immunoassay kit (Thermo Scientific, Waltham, MA) according to the manufacturer’s instructions and recorded on a Hyperfine-ECL detection membrane. The amount of each protein was semi-quantitatively determined as the ratio of GAPDH indicated on each gel.

**Cell counting kit-8 (****CCK-8) assay**

The proliferative ability of the cells was assayed using the CCK-8 assay according to the manufacturer’s instructions (Dojindo Laboratories, Kumamoto, Japan). Cells were seeded at 1×10^3^/well in 96-well plates and medium was changed every other day. Every 24 hours post-transfection for five consecutive days, the cells were treated with CCK-8 solution reagent (10 µl) for 2 h, and the proliferative rate was measured using a plate reader at 450 nm (Bio-Rad, Hercules, CA). All conditions were performed in triplicate.

**Colony-formation assay**

Cells were seeded in 6-well plates with a cell density of 1000 cells per well, and continuously cultured for ten days. Colonies were then fixed with 4% paraformaldehyde for 15 min and visualized by staining for 10 min with 0.5% crystal violet.

**Cell migration assay**

The cell migration ability was tested with Transwell chamber (24-well format) with 8-μm polycarbonate membranes (Corning, NY). Cells were seeded at 2×10^5^ /well in upper chambers with serum-free DMEM and the lower chamber was filled with that with 15% fetal bovine serum. Incubated for 48 h, cells that passed through the membranes were fixed with paraformaldehyde and stained with crystal violet. The number of cells on the lower side of the membrane was counted in five random fields (200×). Each condition was repeated in triplicate.

**Flow cytometry analysis**

Cells were placed in a 6-well plate for 48 h. Then cells were collected and fixed with 70% ethanol in a fixative at -20 °C overnight. Cells were stained with propidium iodide (PI) according to the manufacturer's instructions (Beyotime Biotechnology, Shanghai). The intensity of fluorescence was detected by flow cytometry (B&D system). Each condition was repeated in triplicat.

**Tumor xenografts**

All procedures conformed to the legal mandates and guidelines of the Laboratory Animal Center of Fujian Medical University for the care and maintenance of laboratory animals. 7×10^6^ stable NC or sh-TCP1 MHCC97H cells were subcutaneously injected into the armpit of four- to six-week old nude mice (BALB/c). Six mice were used for each group. The xenograft size was measured every three days. The volume of the xenograft was calculated following the formula: volume = 1/2×length×width^2^. Six weeks after injection, the mice were killed and the tumor specimens were weighed. In order to examine the numbers of lung metastasis nodules, the lungs of mice were embedded in paraffin and serially resected for HE staining. Some of tumor tissues were frozen at −80 °C for IHC analysis.^1^ The mean optical density (%) of TCP1 = IOD/Area.

**WNT7B promoter luciferase constructs**

Genomic DNA from normal blood cells, extracted by Genomic DNA Purification Kit (Promega, Madison, WI), was used as the template for polymerase chain reaction amplification to obtain the putative promoter region of WNT7B (nucleotides -1644 to +77, relative to the translation initiation site) and cloned into pGL4.10-Basic vector (Promega) at *kpn*I and *Hind*III (Thermo Scientific, Waltham, MA) sites to generate pGL4-1721. A series of WNT7B promoter deletion constructs were also made by insertion of the corresponding PCR-generated fragment into the pGL4-Basic plasmid. The pGL4-649 plasmid was used as a template for construction of p53 binding sites mutants (pGL4-mut1, pGL4-mut2) through over-lapping extensive PCR. All primers used for amplification are shown in (Supplementary Table 4) and constructs were confirmed by DNA sequencing.

**Dual-luciferase reporter assay**

Cells were seeded in triplicate into 24-well plates and co-transfected with the indicated Wnt7B promoter reporter vectors, pRL-TK vector (Promega) encoding Renilla luciferase together with the empty vector (pcDNA3.1) or with pcDNA3.1-p53 expression vector. Forty-eight hours after transfection, cells were lysed and their luciferase activities were measured using Dual-Luciferase assay system (Promega) as described previously^2^.

**Chromatin immunoprecipitation assay (****ChIP)**

ChIP was performed using Magna-ChIP TM Chromatin Immunoprecipitation kit (Millipore, Temecula, CA) as described previously.^2^ Bound target DNA fractions were analyzed by qPCR. The primers used are shown in Supplementary Table 4, which amplified a region that spanned nucleotides -253 to +167 (containing the p53 binding sites) of the WNT7B promoter.

**Immunoprecipitation assay (CoIP)**

Cells (1×10^7^) for whole cell lysate were lysed in 800µl ice cold RIPA buffer and incubate at 4°C for 20 minutes. Pellet cellular debris by centrifugation at 10,000 rpm for 10 minutes at 4°C. After determining protein concentration using the Bradford assay, 500µg of the lysate was brought to a final volume of 1ml with PBS, the remaining part was used as the input group for immunoblot analysis. Lysates were precleared with 1μg of appropriate control IgG (Beyotime) and 20 µl of protein A/G Plus-agarose (Santa Cruz) and kept on a rotator for 1 h at 4°C. Lysates were centrifuged (2500rpm for 5min at 4°C) and 2 μg of P53 antibody or corresponding IgG was added to the precleared lysates and incubate at 4°C on a rotating device for 3 to 5 hours. Following incubation, 30µl of protein A/G Plus-agarose was added to each tube and kept on a rotator overnight at 4 °C. Lysates were then centrifuged (2500 rpm for 5 min at 4°C). The pellet fractions were washed 4 times with PBS and then resuspended in 20µl of 1×loading buffer. Samples were immunoblotted with the appropriate antibody as indicated.

**Statistical analysis**

Each cellular experimental group was repeated for at least three times. Statistical significance among different groups in the experiments was tested by ANOVA and unpaired t-test. Overall survival curves were estimated by the Kaplan–Meier method and compared among groups by log-rank test. The Cox proportional hazards regression model was applied to perform univariate and multivariate regression analyses, which calculate the hazard ratios (HRs) and their 95% confidence intervals (95% CIs) for each variable and analyze independent factors affecting prognosis. The results were considered statistically significant when *P*<0.05. The data are expressed as the mean ± SD calculated by GraphPad Prism Version 7 (GraphPad Software Inc., SanDiego, CA).

**Supplemental Figures**


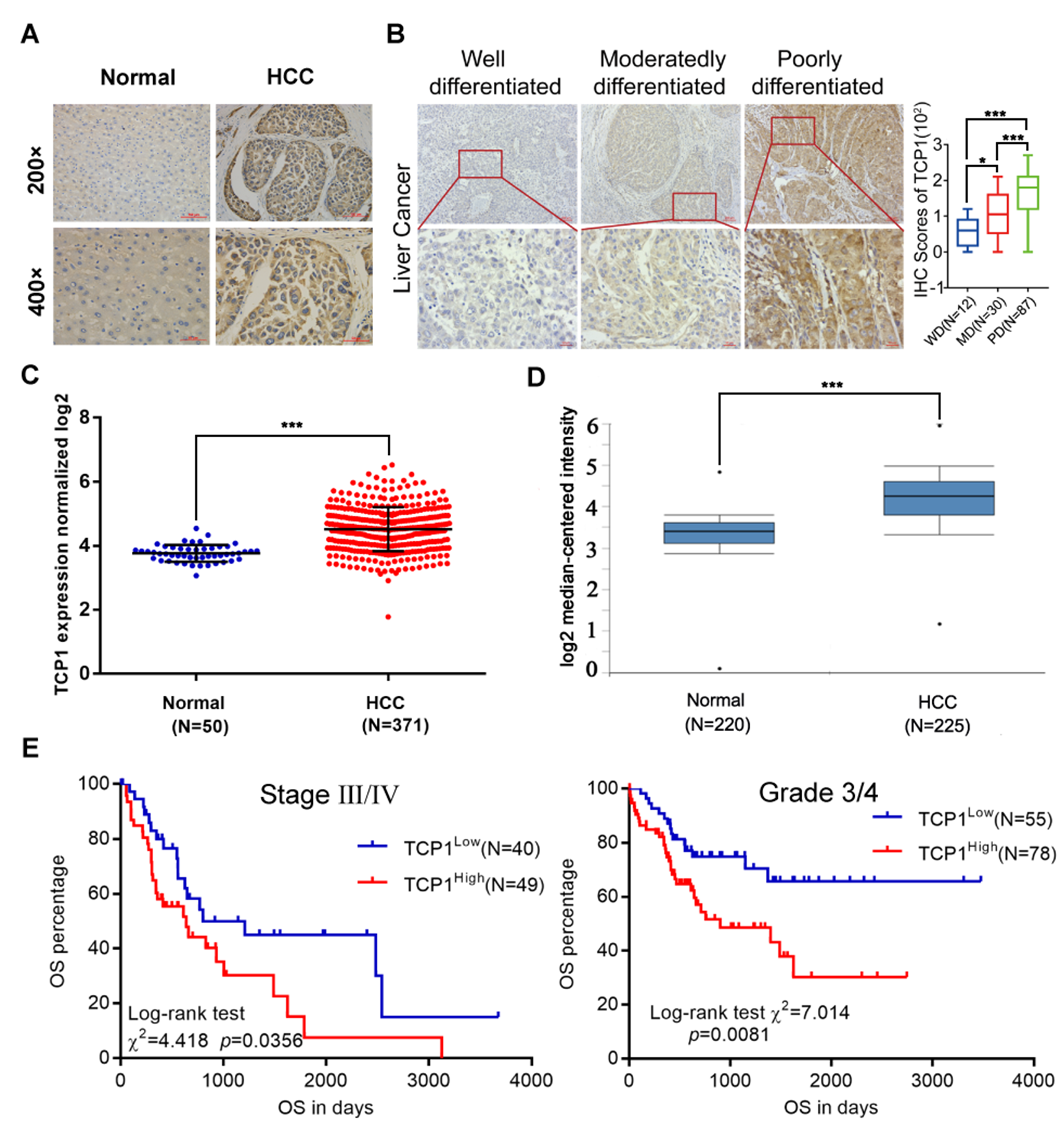


**Fig. S1** **TCP1 expression is elevated in HCC tissues and correlates with a poor prognosis.** (A) Immunohistochemical representative images of TCP1 expression in Dataset 1. (B) Comparison of TCP1 levels in well, moderately or poorly differentiated liver cancer were determined by immunohistochemistry (IHC) analysis of Dataset 2. The horizontal lines in the box plots represent the median, the boxes represent the interquartile range. (C) *TCP1* gene expression from TCGA-LIHC and (D) Oncomine databases. (E) Kaplan-Meier curves of overall survival in patients with primary HCC was performed in stage III/IV and G3/G4 tumors. Data represent the mean ± SEM. * *P*<0.05, *** *P*<0.001.


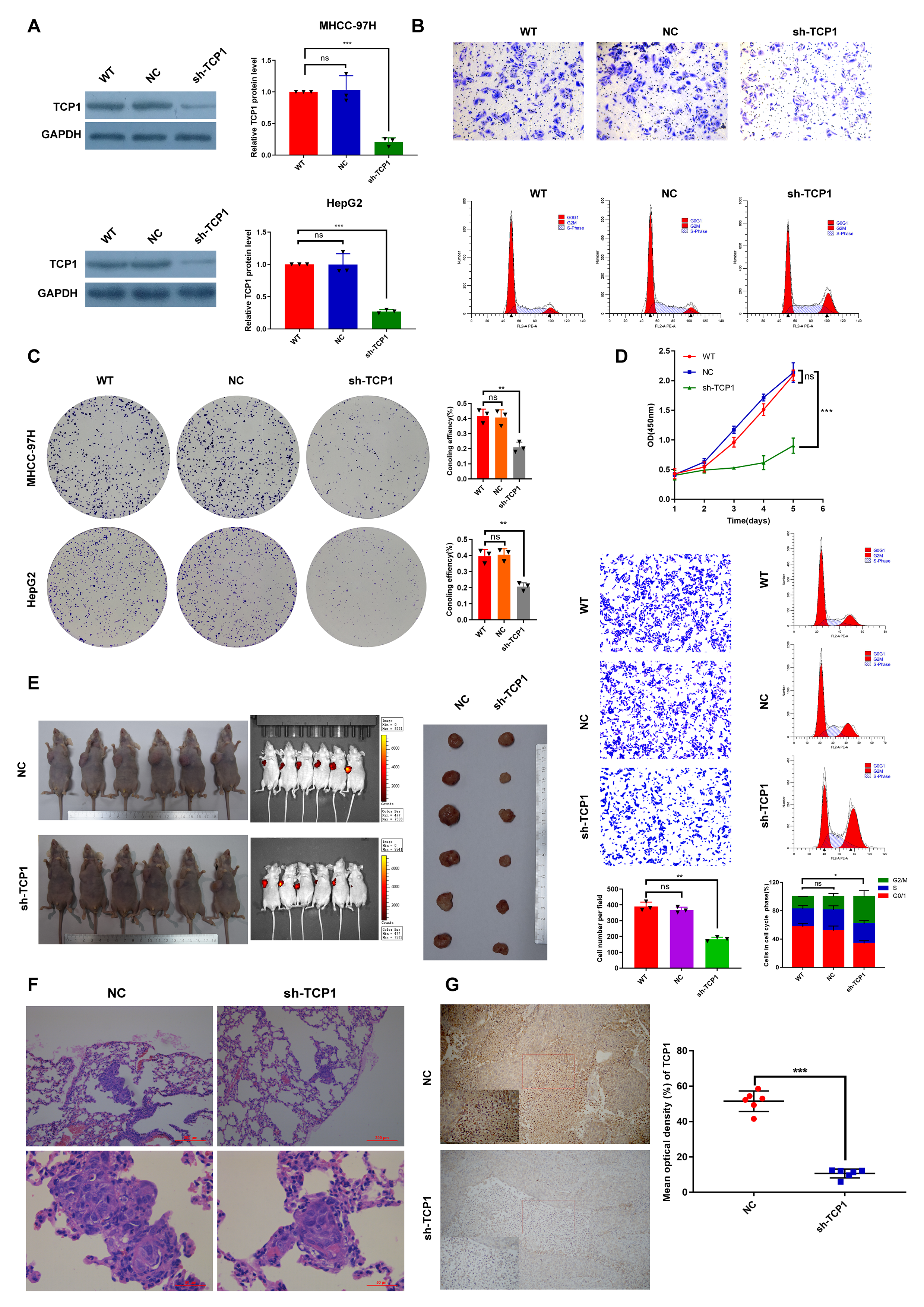


**Fig. S2**  **Knockdown of TCP1 inhibits the proliferation and migration of HCC *in vitro* and *in vivo*.** (A) TCP1 expression in MHCC-97H and HepG2 cells stably transduced with LV-shTCP1 or LV-shNC was analyzed by western blotting. (B) Transwell migration assay and cell cycle analysis of WT (wild type), NC (negative control) and sh-TCP1 (TCP1 knockdown) HCC cells in MHCC-97H. (C) Colony formation assay was measured at 10 days after plating. The right panel is a bar graph for colony-formation efficiency. (D) CCK-8, Transwell migration assay, cell cycle analysis of WT, NC and sh-TCP1 in HepG2 cells. (E) MHCC-97H cells with negative control cells and TCP1 knockdown cells were respectively inoculated subcutaneously into the oxter of six nude mice (n=6), optical *in vivo* imaging and the xenograft tumors. (F) Photographs of the lung metastasis by HE staining. (G) The expression of TCP1 in the tumor tissues of two groups. Data represent the mean ± SEM. * *P*<0.05, ** *P*<0.01, *** *P*<0.001; ns, no significance.


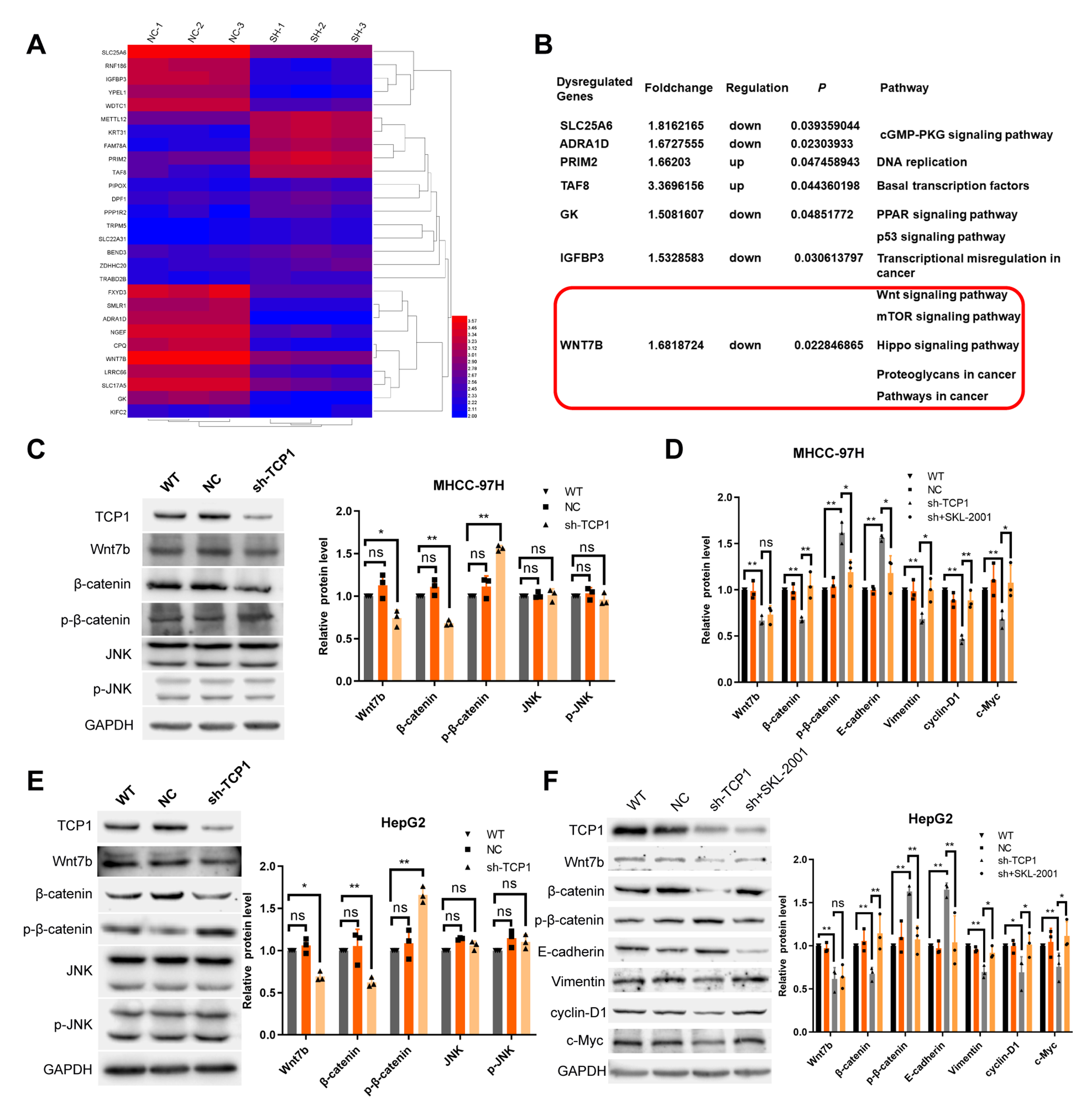


**Fig. S3 Downregulated TCP1 expression inhibits the Wnt/β-catenin signaling pathway of HCC cells through Wnt7b.** (A) The differential gene expression profiles between NC and sh-TCP1 in MHCC-97H cells by microarray-Based gene expression analysis. (B) The significant signaling pathways of the differentially expressed genes. (C) Western blot analysis to detect the protein expression of the Wnt pathway in MHCC-97H cells. (D) Protein levels of the Wnt/β-catenin signaling pathway and it’s downstream proteins related to the epithelial mesenchymal transformation, migration, and cell cycle in MHCC-97H cells (WT, NC, sh-TCP1 and sh-TCP1 in the presence of the SKL2001). (E) Western blot analysis to detect the protein expression of the Wnt pathway in HepG2 cells. (F) Western blot analysis to detect protein levels of the Wnt/β-catenin signaling pathway and it’s downstream proteins in HepG2 cells (WT, NC, sh-TCP1 and sh-TCP1 in the presence of the SKL2001). Data represent the mean ±SEM. * *P*<0.05, ** *P*<0.01, *** *P*<0.001; ns, no significance.


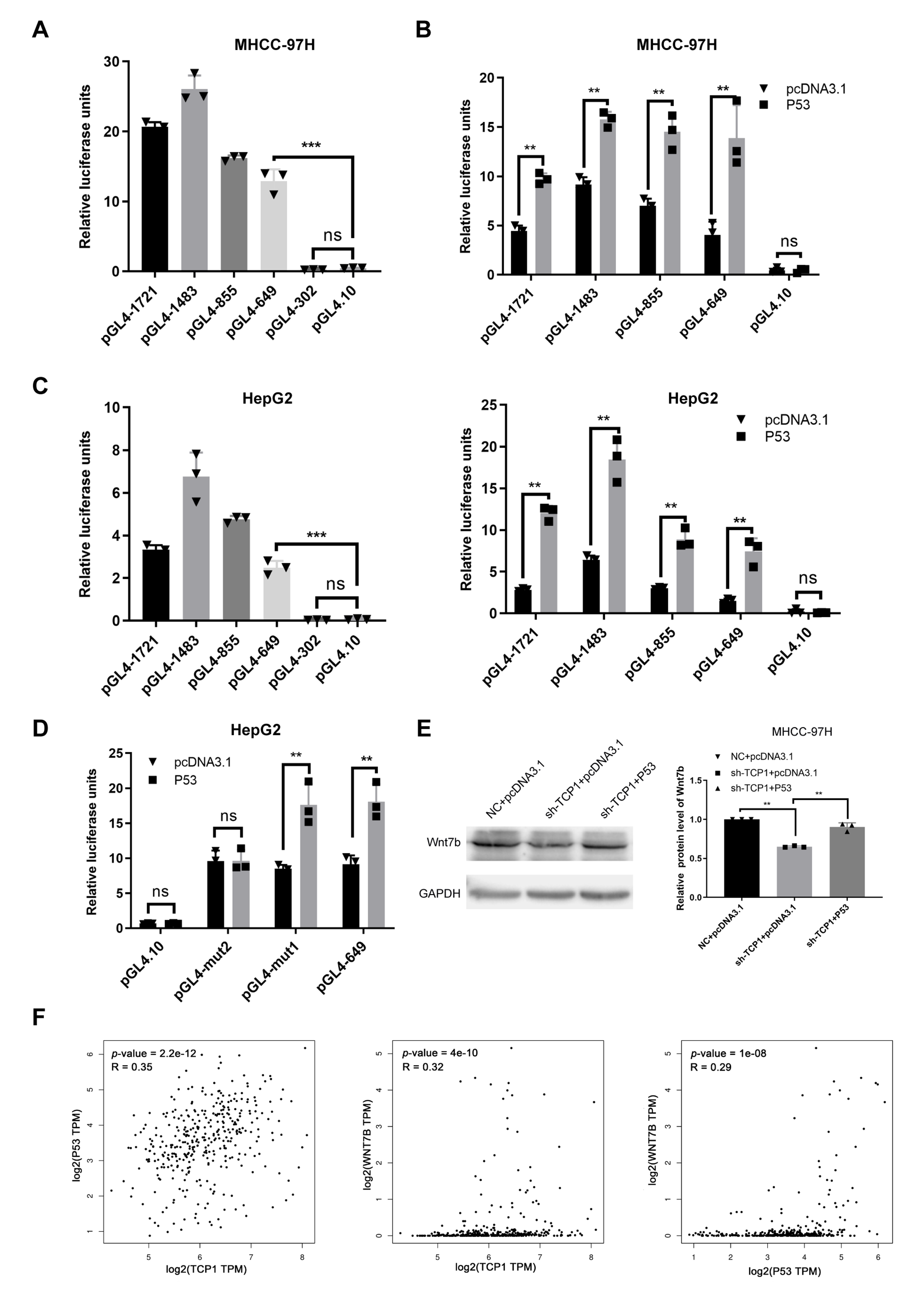


**Fig. S4 TCP1 regulates WNT7B transcription by binding transcription factor P53 in HCC cells.** (A) The activity analysis by dual luciferase reporter in MHCC-97H cells. (C) Overexpression of P53 to detect WNT7B promoter activity. (D) Dual luciferase reporter analysis of HepG2 cells. (E) Western blot analysis for the effect of P53 on Wnt7b expression in MHCC-97H cells with sh-TCP1. (F) The correlations among TCP1, p53 and Wnt7b. Data represent the mean ± SEM. * *P*<0.05, ** *P*<0.01, *** *P*<0.001; ns, no significance.

**Supplementary Tables**

**Supplementary Table 1.** **List of genes affected by TCP1 knockdown and Co-expressed With TCP1***

|  |  | RNA-Seq (sh-TCP1/NC) | | | TCGA LIHC Co-expressed With TCP1 | |
| --- | --- | --- | --- | --- | --- | --- |
| Gene Symbol | Gene Name | Regulation | FC | *P* | Spearman’s Coefficient | *P* |
| PPP1R2 | protein phosphatase 1, regulatory subunit 2 | up | 2.3847 | 0.0161 | 0.4 | 1.1e−15 |
| SENP7 | SUMO1/sentrin specific peptidase 7 | up | 2.266 | 0.015 | 0.33 | 5.1e−11 |
| MAP3K14-AS1 | MAP3K14 antisense RNA 1 | down | 2.1512 | 0.0272 | 0.3 | 4.6e−09 |
| ZDHHC20 | zinc finger, DHHC-type containing 20 | up | 2.0331 | 0.0191 | 0.47 | 3.9e−22 |
| SLC17A5 | solute carrier family 17 member 5 | down | 1.9515 | 0.0386 | 0.47 | 2.4e−21 |
| ODF2 | outer dense fiber of sperm tails 2 | up | 1.9442 | 0.0442 | 0.45 | 1.5e−19 |
| TBC1D19 | TBC1 domain family, member 19 | up | 1.9289 | 0.0178 | 0.34 | 2.1e−11 |
| SLC25A6 | solute carrier family 25 member 6 | down | 1.8162 | 0.0394 | 0.42 | 2e−17 |
| BEND3 | BEN domain containing 3 | up | 1.8121 | 0.0122 | 0.57 | 1.1e−33 |
| WNT7B | wingless-type MMTV integration site family, member 7B | down | 1.6819 | 0.0228 | 0.32 | 4e−10 |
| PRIM2 | primase, DNA, polypeptide 2 | up | 1.662 | 0.0475 | 0.47 | 1.1e−21 |
| SNRPG | small nuclear ribonucleoprotein polypeptide G | up | 1.5795 | 0.0172 | 0.45 | 6.5e−20 |
| YEATS2 | YEATS domain containing 2 | up | 1.5399 | 0.0383 | 0.56 | 8.8e−32 |
| ZNF248 | zinc finger protein 248 | up | 1.5194 | 0.0143 | 0.4 | 2.8e−15 |
| WDR4 | WD repeat domain 4 | up | 1.5169 | 0.0274 | 0.4 | 2.8e−15 |
| PPIL6 | peptidylprolyl isomerase (cyclophilin)-like 6 | down | 1.5105 | 0.0199 | 0.37 | 1.7e−13 |

*****FC>1.5, *P*<0.05, Spearman’s Coefficient>0.3. Abbreviations: FC, fold change; LIHC, liver hepatocellular carcinoma.

**Supplementary Table 2. List of transcription factors predicted by PROMO and JASPAR**

| Model ID | Model name | Score | Relative score | Start | End | Strand | Predicted site sequence |
| --- | --- | --- | --- | --- | --- | --- | --- |
| MA0106.2 | TP53 | 7.604 | 0.806616529392394 | 948 | 962 | -1 | acatgcagggagatg |
| MA0079.1 | SP1 | 9.737 | 0.926505072622137 | 830 | 839 | 1 | ggggcaggga |
| MA0028.1 | ELK1 | 8.858 | 0.906609585159068 | 1370 | 1379 | 1 | caatcggaag |
| MA0154.2 | EBF1 | 8.627 | 0.90588995804989 | 1100 | 1110 | 1 | ggccccagagt |
| MA0024.1 | E2F1 | 8.832 | 0.870252466489806 | 653 | 660 | 1 | cttggcgc |
| MA0466.1 | CEBPB | 2.099 | 0.841941367592672 | 1986 | 1996 | 1 | actttcgcaag |
| MA0095.2 | YY1 | 9.315 | 0.860026814936944 | 1094 | 1105 | 1 | aaggatggcccc |
| MA0105.3 | NFKB1 | 14.171 | 0.955707778165883 | 870 | 880 | -1 | ggggcttcccc |
| MA0007.2 | AR | 4.379 | 0.801241105178428 | 203 | 217 | -1 | cagcacacacagggg |
| MA0084.1 | SRY | 6.572 | 0.829592444937698 | 815 | 823 | 1 | gaaaaccat |
| MA0476.1 | FOS | 5.671 | 0.864573227128281 | 694 | 704 | -1 | cctgattcagg |
| MA0489.1 | JUN | 5.242 | 0.832389492046146 | 695 | 708 | -1 | ctagcctgattcag |
| MA0050.2 | IRF1 | 11.817 | 0.818516119786047 | 1188 | 1208 | -1 | cttggcttccagtttccggtg |
| MA0107.1 | RELA | 11.663 | 0.905627529439086 | 1046 | 1055 | -1 | gggagattcc |
| MA0137.3 | STAT1 | 7.242 | 0.859371298073723 | 1040 | 1050 | 1 | gttgtgggaat |
| MA0526.1 | USF2 | 6.773 | 0.867999859571618 | 439 | 449 | 1 | gccaggtggga |

**Supplementary Table 3. Antibodies used in the present study**

| **Protein name** | **Manufacture (cat.number)** | **Applications (working dilution)** |
| --- | --- | --- |
| TCP1 | Abcam (ab92587) | IB (1:5000), IHC (1:400), IP (1:100) |
| E-cadherin | Affinity (AF0131) | IB (1:1000) |
| Vimentin | Affinity (AF7013) | IB (1:1000) |
| c-Myc | Affinity (AF0358) | IB (1:1000) |
| cyclin-D1 | Affinity (AF0931) | IB (1:1000) |
| β-catenin | SANTA CRUZ (sc-7963) | IB (1:1000) |
| p-β-catenin | Cell Signaling Technology (#9561) | IB (1:1000) |
| JNK | SANTA CRUZ (sc-7345) | IB (1:1000) |
| p-JNK | Cell Signaling Technology (#4668) | IB (1:1000) |
| Wnt7b | Abcam (ab155313) | IB (1:1000) |
| p53 | CST (#9282) | IB (1:1000), IP (1:100) |
| GAPDH | Servicebio (GB11002) | IB (1:5000) |

Abbreviations: IB, immunoblot; IHC, immunohistochemistry; IP, immunoprecipitation; GAPDH, glyceraldehyde3-phosphate dehydrogenase.

**Supplementary Table 4. List of oligonucleotides used in this study***

| **Oligonucleotides** | **Sequences (5’→3’)** |
| --- | --- |
| TCP1-F  TCP1-R  GAPDH-F  GAPDH-R  **WNT7B promoter cloning** | ATGGAAGTTATGAAGATGCTGTTC  TACTCAACTCAAGGTACAAGACAA  AGGGCTGCTTTTAACTCTGGT  TCTCGCTCCTGGAAGATGGTG |
| pGL4-1721(-1644/+77)-F | CGG *GGTACC* GGTTCAGGGATTTGGAGGGATT |
| pGL4-1483(-1406/+77)-F | CGG *GGTACC* GAAAGCACAGAGCAGGCAGGG |
| pGL4-855(-778/+77)-F | CGG *GGTACC* TGGAAGCCAAGTGGCACCG |
| pGL4-649(-572/+77)-F | CGG *GGTACC* CGGGGGGTAGATGCGTTTGTG |
| pGL4-302(-225/+77)-F | CGG *GGTACC* CAGCCTGGGTCAATTCCCC |
| pGL4-1721(-1644/+77)-R | CCC *AAGCTT* AGTGCTCCGAGCTTCACGTAC |
| **Site-directed mutagenesis** |  |
| pGL4-mut1-F | GGCTCGCTTGC**AT**GGGCCGCGAGTC |
| pGL4-mut1-R | GACTCGCGGCCC**AT**GCAAGCGAGCC |
| pGL4-mut2-F | GGCGGCCGACAA**TA**GCGGACACATTGG |
| pGL4-mut2-R | CCAATGTGTCCGC**TA**TTGTCGGCCGCC |
| **ChIP** |  |
| WNT7B-F | GAGCGAGGAGGGCGGGCGGGAGGC |
| WNT7B-R | GCGCAGCCGCCTGAGGCCGTGAGC |
| **P53 Coding sequence** |  |
| **cloning** |  |
| Forward | CCG *CTCGAG* GCCACC ATGGAGGAGCCGCAGTCAGAT |
| Reverse | CCC *AAGCTT* ACAAGAAGTGGAGAATGTCAGT |

* Restriction sites are shown in italics. Mutant bases are underlined. Abbreviations: F, forward primer; R, reverse primer; GAPDH, glyceraldehyde3-phosphate dehydrogenase; ChIP, chromatin immunoprecipitation.

**REFERENCES**

1. Chen L, Guo P, He Y, Chen Z, Chen L, Luo Y, et al. HCC-derived exosomes elicit HCC progression and recurrence by epithelial-mesenchymal transition through MAPK/ERK signalling pathway. Cell Death Dis. 2018;9:513.

2. Chen Z, Tang N, Wang X, Chen Y. The activity of the carbamoyl phosphate synthase 1 promoter in human liver-derived cells is dependent on hepatocyte nuclear factor 3-beta. J Cell Mol Med. 2017;21:2036-45.
